# Maternal micronutrient deficiency and congenital heart disease risk: A systematic review of observational studies

**DOI:** 10.1002/bdr2.2072

**Published:** 2022-08-18

**Authors:** Stuart Mires, Massimo Caputo, Timothy Overton, Clare Skerritt

**Affiliations:** ^1^ Fetal Medicine Unit St Michaels Hospital, University of Bristol Bristol United Kingdom; ^2^ Paediatric Congenital Heart Surgery Bristol Royal Hospital for Children, University Hospitals Bristol and Weston NHS Foundation Trust Bristol United Kingdom; ^3^ Fetal Medicine Unit St Michaels Hospital, University Hospitals Bristol and Weston NHS Foundation Trust Bristol United Kingdom; ^4^ Paediatric General Surgery Bristol Royal Hospital for Children, University Hospitals Bristol and Weston NHS Foundation Trust Bristol United Kingdom

**Keywords:** congenital heart disease, deficiency, micronutrient, risk factor

## Abstract

**Background:**

Congenital anomalies affect over 2% of pregnancies, with congenital heart disease (CHD) the most common. Understanding of causal factors is limited. Micronutrients are essential trace elements with key roles in growth and development. We aimed to investigate whether maternal micronutrient deficiencies increase the risk of fetal CHD through systematic review of published literature.

**Method:**

We performed a systematic review registered at PROSPERO as CRD42021276699. Ovid‐MEDLINE, Ovid‐EMBASE, and Cochrane Library were searched from their inception until September 7, 2021. Case control trials were included with a population of biological mothers of fetuses with and without CHD. The exposure was maternal micronutrient level measured in pregnancy or the postpartum period. Data extraction was performed by one author and checked by a second. Risk of bias assessment was performed according to the Scottish Intercollegiate Guidelines Network guidance. We performed a narrative synthesis for analysis.

**Results:**

726 articles were identified of which 8 met our inclusion criteria. Final analysis incorporated data from 2,427 pregnancies, 1,199 of which were complicated by fetal CHD assessing 8 maternal micronutrients: vitamin D, vitamin B12, folate, vitamin A, zinc, copper, selenium, and ferritin. Studies were heterogenous with limited sample sizes and differing methods and timing of maternal micronutrient sampling. Definitions of deficiency varied and differed from published literature. Published results were contradictory.

**Conclusion:**

There is not enough evidence to confidently conclude if maternal micronutrient deficiencies increase the risk of fetal CHD. Further large‐scale prospective study is required to answer this question.

## 1 INTRODUCTION

Congenital anomalies occur in approximately 1:50 pregnancies. Around 30% are congenital heart disease (CHD), representing the most prevalent congenital anomaly (Public Health England, 2019). CHD is generally defined as a “gross structural abnormality of the heart or intrathoracic great vessels present at birth that is actually or potentially of functional significance (Mitchell et al., 1971).” The most common CHD are ventricular septal defects, atrial septal defects, transposition of the great vessels, patent ductus arteriosus (PDA) and tetralogy of Fallot (Suluba et al., 2020). As PDA is physiological at birth, the EUROCAT congenital anomaly registry classifies PDA as CHD if persistent 6 months following birth or requiring interventional closure (EUROCAT, 2013). The potential causal molecular, genetic and environmental risk factors remain poorly understood. Approximately 20% of cases can be attributed to genetic, chromosomal or teratogenic causes. The remaining 80% are likely multifactorial including genetic and environmental factors (Blue et al., 2012). Congenital anomalies remain a leading cause of neonatal, infant and childhood death (Petrini et al., 2002). Patients with CHD often require multiple and complex surgeries with long term medical therapies and follow up. These can involve significant morbidity, long term mortality and substantial cost (Tennant et al., 2010). Primary prevention of CHD would significantly reduce these patient and clinical burdens (Botto & Correa, 2003). However, this requires a greater understanding of causative factors in CHD.

From Day 18 of human embryonic development, the endocardial progenitor cells form heart tubes which ultimately fuse, fold, loop and septate to form the embryonic heart. The heart beats from Day 22, with the critical sensitive period for cardiovascular development generally recognized as between Weeks 3 and 8 of embryonic development (Moore et al., 2020). These processes are under the control of a number of transcription factors, genes and signaling pathways all influenced by genetic and environmental factors (Suluba et al., 2020).

Micronutrients are essential trace elements and vitamins (Shenkin, 2006). Vitamin and micronutrient supplementation in pregnancy is recommended by the Royal College of Obstetricians and Gynecologists (RCOG) and National Institute for Health and Care Excellence (NICE) in the United Kingdom. These include folate before pregnancy and throughout the first 12 weeks predominantly to reduce the risk of neural tube defects and vitamin D to improve growth and reduce deficiency in neonates (NICE, 2014, 2017; RCOG, 2014). Micronutrients play essential roles in intracellular and cellular signaling and growth, and as such facilitate development. Deficiencies may arise secondary to dietary and physiological factors. Animal studies assessing deficiencies have shown increased rates of congenital anomalies (McArdle & Ashworth, 1999). Studies of CHD in animal models show maternal micronutrient deficiencies including iron and vitamin A lead to increased rates in offspring (Joshi et al., 2020; Kalisch‐Smith et al., 2021). Assessment in these models suggests alterations in signaling pathways including retinoic acid signaling or epigenetic modifications potentially drive pathogenesis. This can be prevented through micronutrient administration (Joshi et al., 2020; Kalisch‐Smith et al., 2021). Systematic review and meta‐analyses of folate and multivitamin supplementation in pregnancy and the periconceptual period have illustrated reductions in CHD and cardiovascular anomalies, neural tube defects, limb defects, orofacial clefts and urinary tract anomalies in human fetuses (Botto et al., 2004; Feng et al., 2015; Ingrid Goh et al., 2006). However, the included observational studies largely assess supplementation and do not ascertain maternal micronutrient levels.

To our knowledge, a systematic review of maternal micronutrient deficiencies in CHD has not been undertaken previously. We therefore aim to critically appraise available literature to address the question of whether maternal micronutrient deficiencies increase the risk of fetal CHD.

## 2 METHODS

### 2.1 Eligibility criteria

#### 2.1.1 Study design

The overarching question addressed in this systematic review was do maternal micronutrient deficiencies increase the risk of fetal CHD? Observational studies were included. It was not anticipated that data from randomized control trials or controlled trials would be available. Case series and case reports were excluded due to high risk of bias.

#### 2.1.2 Population

The target population was pregnant biological mothers of fetuses. Studies including surrogate mothers or pregnancies with egg donation were excluded due to the potential influence of micronutrient levels in the donor or surrogate. The exposure was maternal micronutrient level measured through serum or hair analysis in pregnancy or the postpartum period. Micronutrients in included studies were vitamin A, vitamin B 12, vitamin D, folate, iron, selenium, copper, and zinc.

#### 2.1.3 Outcomes

The outcome assessed was a fetus, neonate or child with CHD. CHD was diagnosed antenatally though fetal echocardiography or postnatally through echocardiography, clinical assessment, cardiac catheterization or surgery.

### 2.2 Information sources and search strategy

This systematic review protocol was registered at PROSPERO (registration number CRD42021276699). We used the Preferred Reporting Items for Systematic review and Meta‐Analysis (PRISMA) 2020 checklist to develop this systematic review (PRISMA, 2020). A systematic literature search of electronic databases MEDLINE (via OVID), EMBASE (via OVID) and the Cochrane library was conducted to identify relevant studies published between the beginning of each database and September 7, 2021 without language restrictions. We also manually screened reference lists of included studies to identify any additional relevant literature. A detailed search strategy for each database is outlined in Figure S1. The search strategy was developed around the MeSH descriptors: exp[heart defects, congenital], [micronutrients], [vitamins], [deficiency diseases], exp[avitaminosis]; and the key terms: [congenital heart disease*.tw.], [congenital cardiac disease*.tw.], [congenital heart anomal*.tw.], [congenital cardiac anomal*.tw.], [maternal vitamin*.tw.], [maternal micronutrient*.tw.], [maternal zinc.tw.], [maternal copper.tw.], [maternal iron.tw.] and [maternal fol*.tw.].

### 2.3 Study selection, data extraction and risk of bias assessment

Two authors (SM and CS) independently screened the titles and abstracts to exclude publications that did not meet the inclusion criteria. Any discrepancies were discussed. After selecting studies for inclusion, data extraction was performed by SM and checked by CS into a prior agreed form. Data extracted included author, year of publication, country of origin, study design, inclusion and exclusion criteria, case and control matching data, micronutrients analyzed, source and timing and CHD diagnosis and analysis. Information on confounding variables and adjusted data was collected. Risk of bias assessment was assessed by SM and CS utilizing the Scottish Intercollegiate Guidelines Network (SIGN) case control study checklist and associated guidance notes (SIGN, 2012). This facilitates assessment of subject selection, exposure assessment, confounding and statistical analysis. Studies are assessed overall as high quality (++), acceptable (+) or unacceptable. Unacceptable studies were excluded from analysis due to high risk of bias.

### 2.4 Data synthesis

Results from studies were summarized with a narrative synthesis including summary tables due to the diversity of the included studies. Quantitative synthesis of data was not performed due to the heterogeneity of studies. Analysis was stratified by micronutrient subgroups and micronutrient levels and crude and adjusted odds ratios (OR) were presented. Where possible, if not presented in the primary study, crude ORs were calculated by the study authors. Micronutrients levels were converted into the same unit for each study to allow for direct comparison. Published micronutrient reference ranges in pregnancy were used to define deficiency to further aid study comparisons (Cunningham, 2010).

## 3 RESULTS

### 3.1 Literature search

**FIGURE 1:**
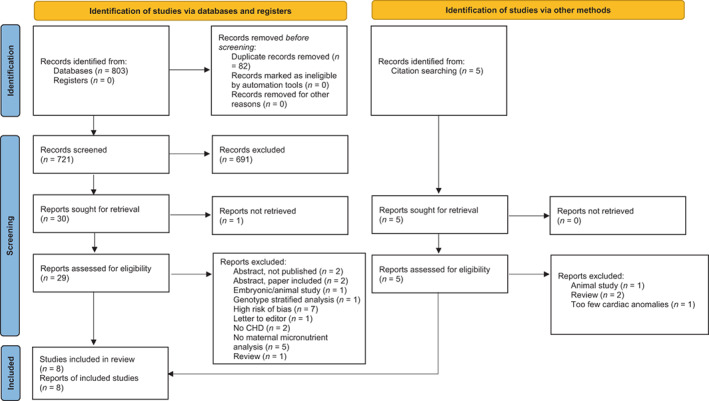
PRISMA 2020 flow diagram of study identification, screening, inclusion, and exclusion from Page et al. (2021)

A PRISMA flow chart of included studies is seen in Figure 1. Seven hundred and twenty‐six unique citations were identified after an initial database and reference search. Of these, 691 were excluded after title and abstract screening. The full text of 34 articles were reviewed, with 1 report not retrieved due to journal inaccessibility. A further 26 articles were excluded with reasons outlined in Figure 1, and 8 articles included in the systematic review (Dilli et al., 2018; Hobbs et al., 2005; Hu et al., 2014; Koster et al., 2018; Mokhtar et al., 2019; Ou et al., 2017; Verkleij‐Hagoort et al., 2006; Yang et al., 2020).

### 3.2 General characteristics of studies

The characteristics of the studies included in the review are summarized in Table S1. The review included data from 2,427 pregnancies, of which 1,199 had fetuses affected by CHD. Data was available on maternal levels of 8 micronutrients across studies: vitamin D, vitamin B12, folate, vitamin A, zinc, copper, selenium and ferritin. Studies originated from 5 countries: Turkey, Netherlands (2), United States, China (3), and Egypt. Studies were published in English between 2005 and 2020.

In all but 1 study, maternal micronutrient testing was through venous blood analysis of serum or plasma. One study including copper and zinc micronutrients performed testing of maternal hair. Of the six studies reporting timing of sampling, four assessed micronutrient levels postnatally. Two studies assessed levels during pregnancy between 17 weeks and delivery. In three studies CHD diagnosis was performed antenatally, four studies performed postnatal diagnosis and one study included both with cases identified through a state registry. Each study had varying proportions of diverse CHD.

The range of included studies with heterogenous micronutrient assessment, sampling technique, sample timing and CHD diagnosis and subtypes precluded quantitative synthesis of data.

### 3.3 Micronutrient subgroups

Table 1 summarizes differences in micronutrient levels between cases and control groups for the seven micronutrients included and ORs where calculated. Table 2 summarizes published reference ranges for micronutrients for nonpregnant and second trimester pregnant women (Cunningham, 2010).

**TABLE 1:** Assessment of micronutrient levels and odds ratios in included studies (NR ‐ not recorded) (Dilli et al., 2018; Hobbs et al., 2005; Hu et al., 2014; Koster et al., 2018; Mokhtar et al., 2019; Ou et al., 2017; Verkleij‐Hagoort et al., 2006; Yang et al., 2020)

Micronutrient	Raw data		Odds ratios	Effect direction
Vitamin D	Dilli et al. Mean ± *SD* nmol/L	Cases: 26.7 ± 18.0 Controls: 34.4 ± 16.7 *p* = .02	Unable to calculate with available data	Lower levels of vitamin D in cases than controls No definition of reference ranges for deficiency and therefore OR not calculated
	Koster et al. Median (IQR) nmol/L	Cases: 57.8 (41.6–78.7) Controls: 65.8 (40.5–86.6) *p* = .02	Vitamin D deficient < 50 nmol/L, moderate 50–75 nmol/L. Utilizing adequate vitamin D group (>75 nmol/L) as reference Crude OR for CHD in offspring: Deficient 1.63 (1.15–2.32) *p* = .006 Moderate 1.60 (1.12–2.28) *p* = .006 aOR (age, BMI, ethnicity, smoking) for CHD in offspring: Deficient 1.82 (1.25–2.66) *p* = .002 Moderate 1.58 (1.11–2.26) *p* = .002	Lower levels of vitamin D in cases than controls Crude and adjusted OR suggest increased odds of CHD in offspring with vitamin D deficiency and moderate levels
	Mokhtar et al. Median (IQR) nmol/L	Cases: 26 (10–NR) Controls: 37 (16–76) *p* = .002	Vitamin D deficient < 50 nmol/L, moderate 50–75 nmol/L. Utilizing adequate vitamin D group (>75 nmol/L) as reference^a^ Crude OR for CHD in offspring: Deficient 2.48 (0.58–10.70) *p* = .22 Moderate 1.09 (0.20–6.01) *p* = .92	Lower levels of vitamin D in cases than controls Crude OR does not provide evidence for increased odds of CHD in offspring with vitamin D deficiency or moddilli erate levels
Ferritin	Yang et al. Median (IQR) μg/L	Cases: 14.3 (9.7–30.3) Controls 21.6 (14.1–39.9) *p* = .003	Mixed linear regression model with control group as reference. No definitions of deficiency Unadjusted expB: Ferritin 0.71 (0.56–0.89) *p* = .003 Adjusted expB (maternal age, gestational age, residence, education, occupation, parity, passive smoking, folate supplement): Ferritin 0.69 (0.54–0.88) *p* = .001	Lower levels of ferritin in cases than controls Unadjusted and adjusted expB suggest lower levels of serum ferritin in case group prior to delivery than control group
Copper	Hu et al. Median (IQR) μg/g	Cases: 12.79 (8.96–17.25) Controls: 8.77 (7.56–10.91) *p* < .001	Utilized control group 5th and 95th centiles as reference ranges. Used 5th–95th group as reference Crude OR for CHD in offspring: Copper <5th 0.36 (0.1–1.36) Copper >95th 5.38 (2.64–10.95) aOR (age, maternal residence, folic acid supplement, previous pregnancy) for CHD in offspring: Copper <5th 0.63 (0.15–2.73) Copper >95th 5.7 (2.58–12.61)	Higher levels of copper in cases than controls Crude OR and aOR does not provide evidence for increased odds of CHD in offspring with low copper. Suggest high copper levels in case group compared to controls
	Ou et al. Median (IQR) μg/L	Cases: 838.38 (778.90–936.98) Controls: 896.56 (817.62–979.44) *p* = .02	Utilized control group tertiles as reference ranges. Used low tertile as reference. Multivariable multielement logistic regression aOR (age, parity, education, newborn gender, migration, folic acid/multivitamin, smoking, pre‐pregnancy BMI, gestation sample) for CHD in offspring: Copper middle tertile 0.57 (0.22–1.47) Copper high Tertile 0.77 (0.31–1.89) Utilized control group tertiles as reference ranges. Used middle tertile as reference.^a^ Crude OR for CHD in offspring: Copper low Tertile 1.91 (0.996–3.661) *p* = .0514	Lower levels of copper in cases than controls aOR does not provide evidence for increased odds of CHD in offspring with high copper Crude OR does not provide evidence for increased odds of CHD in offspring with low copper
Selenium	Ou et al. Median (IQR) μg/L	Cases: 172.90 (153.87–192.23) Controls: 186.47 (172.45–207.34) *p* < .001	Utilized control group tertiles as reference ranges. Used low tertile as reference. Multivariable multielement logistic regression aOR (age, parity, education, newborn gender, migration, folic acid/multivitamin, smoking, pre‐pregnancy BMI, gestation sample) for CHD in offspring: Selenium middle tertile 0.55 (0.22–1.38) Selenium high tertile 0.25 (0.08–0.77) Utilized control group tertiles as reference ranges. Used middle tertile as reference^a^ Crude OR for CHD in offspring: Selenium low tertile 2.17 (1.15–4.11) *p* = .0171	Lower levels of selenium in cases than controls aOR suggest decreased odds of CHD in offspring with higher selenium Crude OR suggest increased odds of CHD in offspring with lower selenium
Zinc	Dilli et al. Mean ± *SD* μg/DL	Cases: 86.0 ± 15.3 Controls: 69.4 ± 15.1 *p* < .001	Unable to calculate with available data	Higher levels of zinc in cases than controls No definition of reference ranges for deficiency and therefore OR not calculated
	Hu et al. Median (IQR) μg/g	Cases: 180.01 (144.59–264.39) Controls: 166.44 (142.02–253.99) *p =* 0.536	Utilized control group 5th and 95th centiles as reference ranges. Used 5th–95th group as reference. Crude OR for CHD in offspring: Zinc <5th 0.67 (0.24–1.87) Zinc >95th 0.50 (0.17–1.46) aOR (age, maternal residence, folic acid supplement, previous pregnancy) for CHD in offspring: Zinc <5th 0.69 (0.23–2.04) Zinc >95th 0.56 (0.16–1.91)	No difference in zinc levels between cases and controls. Crude OR and aOR does not provide evidence for increased odds of CHD in offspring with high or low zinc.
Vitamin A	Dilli et al. Mean ± *SD* mg/L	Cases: 692.6 ± 341.4 Minimum 0.55 maximum 1,060 Controls: 749.5 ± 338.6 Minimum 0.15 maximum 1,690 *p* = .38	Unable to calculate with available data	No difference in vitamin A levels between cases and controls No definition of reference ranges for deficiency and therefore OR not calculated
Vitamin B12	Dilli et al. Mean ± *SD* ng/L	Cases: 249.7 ± 105.8 Controls: 251.4 ± 87.6 *p* = .93	Unable to calculate with available data	No difference in vitamin B12 levels between cases and controls No definition of reference ranges for deficiency and therefore OR not calculated
	Verkleij Hagoort et al. Median (min–max) ng/L	Cases: 356.46 (111.14–815.93) Controls: 336.13 (120.62–1,053.12) *p* > .05	Utilized control group < 10th (159 pmol/L) as deficiency definition. Used > 10th group as OR reference Crude OR for CHD in offspring: B12 < 10th 1.8 (0.8–4)	No difference in vitamin B12 levels between cases and controls Crude OR does not provide evidence for increased odds of CHD in offspring with low B12
	Hobbs et al. Median (min–max) ng/L	Cases: 418.20 (94.21–957.30) Controls: 454.50 (100.72–1,045.0) Unadjusted *p* = .2569 Adjusted *p* = 0.3953 (age, race, education, income, cigarette use, alcohol consumption, vitamin intake, caffeine intake, interval pregnancy to testing)	No reference ranges. OR for association between case or control status and log transformed B12 concentration aOR (other biomarkers—folate, homocysteine, methionine, SAH, SAM, adenosine), education, cigarette use, interval pregnancy to testing for CHD in offspring: B12 1.35 (0.58–3.15) *p* = 0.4875	No difference in vitamin B12 levels between cases and controls aOR does not provide evidence for increased odds of CHD in offspring in relation to B12
Folate	Dilli et al. Mean ± *SD* nmol/L	Serum folate Cases: 22.27 ± 13.18 Controls: 22.27 ± 10.45 *p* = 0.94	Unable to calculate with available data	No difference in serum folate levels between cases and controls No definition of reference ranges for deficiency and therefore OR not calculated
	Verkleij Hagoort et al. Median (min–max) nmol/L	Serum folate Cases: 15 (6.2–27.8) Controls: 14.1 (6.4–40.3) *p* > .05 RBC folate Cases: 646 (299–1,343) Controls: 630 (163–1,549) *p* > .05	Utilized control group < 10th as reference ranges (<10.1 nmol/L serum, <431 nmol/L RBC). Used > 10th group as OR reference Crude OR for CHD in offspring: Serum folate<10th 1.3 (0.5–3.1) RBC folate<10th 1.1 (0.4–2.6)	Higher levels of serum folate in cases than controls. However, not significant to 5% level when adjusted aOR does not provide evidence for increased odds of CHD in offspring in relation to plasma folate
	Hobbs et al. Median (min–max) mg/L	Plasma folate Cases: 9.6 (1.86–30.23) Controls: 10.66 (3.55–40.12) Unadjusted *p* = 0.0174 Adjusted *p* = 0.1681 (age, race, education, income, cigarette use, alcohol consumption, vitamin intake, caffeine intake, interval pregnancy to testing)	No reference ranges. OR for association between case or control status and log transformed plasma folate concentration aOR (other biomarkers—Folate, homocysteine, methionine, SAH, SAM, adenosine), education, cigarette use, interval pregnancy to testing for CHD in offspring: Plasma folate 0.89 (0.41–1.96) *p* = 0.7781	No difference in folate levels between cases and controls aOR does not provide evidence for increased odds of CHD in offspring in relation to B12

a OR not calculated in original paper, therefore calculated by authors.

**TABLE 2:** Published reference ranges for micronutrients (Cunningham, 2010)

Micronutrient (unit)	Not pregnant	Second trimester
Vitamin A (mg/L)	0.2–1	0.35–0.44
Vitamin B12 (ng/L)	279–966	130–656
Vitamin D (nmol/L)	34.94–199.68	24.96–54.91
Ferritin (μg/L)	10–150	2–230
Serum folate (nmol/L)	12.24–40.79	1.81–54.38
RBC folate (nmol/L)	339.90–1,019.70	213.00–1876.25
Copper (μg/L)	700–1,400	1,650–2,210
Selenium (μg/L)	63–160	75–145
Zinc (μg/dL)	75–120	51–80

*Note*: NR ‐ not recorded.

#### 3.3.1 Vitamin D

Three studies assessed vitamin D levels between cases and controls (Dilli et al., 2018; Koster et al., 2018; Mokhtar et al., 2019). All three identified significantly lower levels of vitamin D in cases when compared to control samples. Two studies specified timing of maternal blood sampling (Dilli et al., 2018; Koster et al., 2018). The mean vitamin D levels in both cases and controls reported by Dilli et al., when compared to published reference ranges were classified as deficient. ORs could not be calculated for this study from available data. Median vitamin D levels reported by Koster et al. for cases and controls were in the normal range for published reference ranges. This robust study design with a large sample size suggested an increased adjusted odds of CHD in vitamin D deficiency (1.82 [1.25–2.66]; *p* = .002). Confounders adjusted for included age, body mass index (BMI), ethnicity and smoking. However, deficiency was defined as <50 nmol/L, a higher value than in published literature (lower reference range 34.94 nmol/L). Mokhtar et al. did not define timing of maternal blood sampling and as such vitamin D levels could not be compared to published reference ranges. Crude OR suggested no evidence for increased odds of CHD in vitamin D deficiency, defined as <50 nmol/L. However, this study had a small sample size of 50 cases and controls with odds ratios not adjusted for potential confounders.

#### 3.3.2 Vitamin B12

Three studies assessed vitamin B12 levels between cases and controls (Dilli et al., 2018), with all three studies showing no significant difference in B12 levels. The mean vitamin B12 levels reported by Dilli et al. in cases and controls were both deficient when compared to published reference ranges. ORs could not be calculated from available data. The median vitamin B12 levels reported by Verkleij‐Hagoort et al. (2006) in cases and controls were both in the normal range when compared to published reference ranges. Crude OR did not suggest an increase in odds of CHD in mothers with B12 deficiency. However, deficiency was defined as <159 ng/L which is significantly lower than published definition of deficiency. Therefore, this challenges this finding. The median vitamin B12 levels reported by Hobbs et al. (2005) in cases and controls were both in the normal range when compared to published reference ranges. Adjusted OR did not provide evidence of increased odds of CHD in lower vitamin B12. However, no references for odds calculation were stipulated.

#### 3.3.3 Folate

Three studies assessed folate levels between cases and controls, with all three studies showing no significant difference in folate levels (Dilli et al., 2018). ORs could not be calculated from available data for Dilli et al. OR presented by Verkleij‐Hagoort et al. and Hobbs et al. did not suggest an increase in odds of CHD in mothers with a lower folate. Dilli et al. and Verkleij‐Hagoort et al. assessed serum folate levels, with the mean or median level for both cases and controls within the normal range when compared to published reference ranges. No available published reference values are available for plasma folate assessed by Hobbs et al.

#### 3.3.4 Vitamin A

One study assessed vitamin A levels between cases and controls with no significant difference between the two groups (Dilli et al., 2018). The range of vitamin concentrations for both cases and controls were large, with the mean values above the upper end of published reference values for both cases and controls. Assessment was done in a small sample of 50 cases and 50 controls. ORs could not be calculated from available data.

#### 3.3.5 Zinc

Two studies assessed zinc levels between cases and controls (Dilli et al., 2018; Hu et al., 2014). Hu et al. (2014) assessed hair concentrations of zinc. Analysis of hair did not identify any significant difference in levels of zinc between cases and controls. Crude and adjusted OR did not provide evidence for increased odds of CHD in offspring with high or low zinc. Published reference values for hair concentrations are not available and therefore control group 5th and 95th centiles were used as references for deficiency and excess. Dilli et al. assessed serum concentrations and identified significantly higher levels of zinc in cases than controls. ORs could not be calculated for this study from available data. When compared to published reference ranges, mean zinc concentration of the control group was deficient while the case group was in the normal range. Furthermore, a small sample of 50 cases and 50 controls were assessed. These heterogenous studies test different maternal sample sites with contradictory findings.

#### 3.3.6 Copper

Two studies assessed copper levels between cases and controls (Hu et al., 2014; Ou et al., 2017). Hu et al. assessed hair concentrations of copper, with significantly higher levels of copper in cases than controls. ORs adjusted for age, maternal residence, folic acid supplementation and previous pregnancy suggested an increased odds of CHD in higher copper hair concentrations (5.7 [2.58–12.61]). There was no evidence of increased odds of CHD in lower copper hair concentrations. This study was well designed, utilizing the 5th and 95th centiles of the control group copper levels as references for deficiency and excess due to lack of published reference values for hair concentrations. Ou et al. assessed plasma concentrations of copper, with significantly lower levels of copper in cases than controls. However, crude OR did not provide evidence of increased odds of CHD in offspring of mothers with low copper. When plasma values were compared to published reference ranges, the median level in both cases and controls was deficient. The tertiles of control group data were utilized as reference for ORs and therefore, assessment of CHD risk relative to deficiency is limited. These heterogenous studies test different maternal sample sites with contradictory findings.

#### 3.3.7 Selenium

One study assessed selenium levels between cases and controls (Ou et al., 2017). Lower levels of selenium were present in cases than in controls, with decreased adjusted odds of CHD in offspring with higher selenium levels. However, both cases and controls had higher median selenium levels than normal reference values, with the tertiles of control group utilized as references for OR calculation. While well designed, this limits assessment of CHD risk relative to selenium deficiency.

#### 3.3.8 Ferritin

One study assessed ferritin levels between cases and controls (Yang et al., 2020), with significantly lower levels of ferritin present in cases. However, both cases and controls had ferritin levels within the normal range when compared to published reference ranges. Mixed linear regression modeling adjusting for maternal age, gestational age, residence, education, occupation, parity, passive smoking and folate supplementation suggested lower levels of serum ferritin in cases than controls. There was no definition or assessment of ferritin (iron) deficiency in analysis. Furthermore, this was a small sample of 50 cases and 100 controls where biochemical analysis was performed.

### 3.4 Confounding variable assessment

**FIGURE 2:**
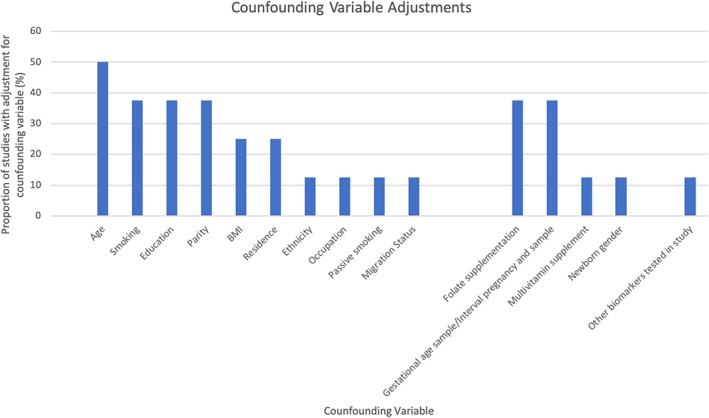
Confounding variables that were adjusted for in included studies, proportion of studies

Figure 2 summarizes the proportion of studies which accounted for individual confounding variables in their analysis of odds of CHD. Age was the most frequent confounder accounted for in 50% of studies, followed by maternal smoking status, maternal education, parity, folate supplementation and timing of maternal micronutrient sampling in 37.5% of studies. It is therefore clear that across the included studies, unaccounted for confounding variables may bias analyses presented.

## 4 DISCUSSION

### 4.1 Main findings and interpretation

Assessing the eight micronutrients included in this systematic review, there is not enough evidence to confidently determine if maternal micronutrient deficiency increases the risk of CHD in fetuses. This is exemplified in the small sample sizes included from heterogenous studies, reporting contradictory findings. Studies included were all of case control design. Data from randomized controlled or controlled trials was not available.

Included studies suggested lower levels of vitamin D, selenium and ferritin in cases than controls; no difference in levels of vitamin B12, folate and vitamin A; and contradictory findings for zinc and copper (Dilli et al., 2018; Hobbs et al., 2005; Hu et al., 2014; Koster et al., 2018; Mokhtar et al., 2019; Ou et al., 2017; Verkleij‐Hagoort et al., 2006; Yang et al., 2020). Deficiency is defined as “a shortage of a substance (as vitamins) necessary to health” (Merriam‐Webster, 2021). Pregnancy is a state of altered physiology and metabolism which can influence laboratory test value ranges (Cunningham, 2010). Reference values are rarely reported for pregnancy. In this systematic review, we compared median or mean micronutrient levels for cases and controls to published reference ranges established by systematic review of literature utilizing the 2.5–97.5 centiles as a normal range (Cunningham, 2010). When compared to these published reference ranges, mean or median values reported for cases and controls in studies were often both deficient or both in the normal range. For vitamin D and vitamin B12 where deficiency was assessed by ORs, deficiency threshold defined in the studies differed from published reference ranges, limiting these findings (Koster et al., 2018; Mokhtar et al., 2019; Verkleij‐Hagoort et al., 2006). Furthermore, published reference ranges are only available for blood samples and not hair (Hu et al., 2014). One study of copper and zinc utilized hair sampling as opposed to blood sampling (Hu et al., 2014). While well designed, it is unclear whether control group references are representative of the population. This could contribute to the contradictory findings in these micronutrients. Therefore, across the eight micronutrients studied, true assessment of deficiency has not been performed and limits conclusions drawn.

Numerous factors influence micronutrient levels including BMI, age, ethnicity and a number of lifestyle factors such as smoking (Damms‐Machado et al., 2012; Kant & Graubard, 2007; Preston, 1991; Richard & Roussel, 1999). Of the eight papers included, five adjusted for confounders in their analysis (Hobbs et al., 2005; Hu et al., 2014; Koster et al., 2018; Ou et al., 2017; Yang et al., 2020). However, as Figure 2 illustrates, adjustments for confounders were limited with factors such as ethnicity only being recognized in 12.5% of studies. Furthermore, pregnancy and gestational age are known to influence blood micronutrient levels (Cunningham, 2010). This is particularly important for systematic review given the heterogeneity of timing of sampling in the included studies. Of the included studies, four sampled postnatally, two antenatally and two did not specify timing. However, given the critical sensitive period for cardiac development of 3–8 weeks, nutrition in the periconceptual period is likely most relevant (Moore et al., 2020). Studies assessing micronutrient levels and dietary patterns suggest that postnatal assessment of a number of micronutrients including vitamin B12, folate and zinc represents that of the periconceptual period (Cucó et al., 2006; Devine et al., 2000; van Driel et al., 2010). However, sample sizes were small, with only 30 patients in the direct study of micronutrient levels (van Driel et al., 2010). Furthermore, this was performed in fertility patients, potentially unrepresentative of the general population (van Driel et al., 2010). Therefore, the sample timings in included studies may not accurately represent periconceptual and early pregnancy levels, limiting findings.

Despite these limitations, there remains biological and physiological plausibility for specific micronutrient deficiencies being associated with fetal CHD. Micronutrients are known to play essential roles in cellular signaling, growth and development (McArdle & Ashworth, 1999). Vitamin D deficiency is a significant public health issue, with prevalence reported in 40–80% of the pregnant population. Maternal and fetal concentrations increase by up to 200% in the first trimester, with vitamin D acting on gene expression to influence long term health (Kaludjerovic & Vieth, 2010). Animal models have demonstrated knockdown of the vitamin D receptor is characterized by CHD (Kwon, 2016). Selenium performs biological function within selenoproteins, involved in vast physiological processes including protecting cells from oxidative stress (Labunskyy et al., 2014). Iron deficiency anemia is common in pregnancy, affecting approximately 20% of women in developed countries (Allen, 2000). Animal modeling of iron deficiency and defects in the transferrin receptor have been associated with neural tube and cardiovascular defects in animal models (Andersen et al., 2006; Hoyle et al., 1996). These associations and biological roles stress the importance of future research in this field.

### 4.2 Strengths and limitations

This study was the first to address this topic and performed a robust review with clear inclusion criteria and population definitions. Further comparison to established published reference ranges strengthens the findings. This facilitates objective comparison across studies and illustrates the heterogeneity of results. The use of recognized reference ranges defining deficiency is essential in future studies in this field. However, there are limitations. The inclusion of observational studies introduces uncertainty through their inherent limitations including susceptibility to bias and confounding. Inclusion and performance of such studies is necessitated in this subject area where controlled studies will not be available. Therefore, comparisons of case and control groups with full recognition of confounders in analysis is key for future research. The heterogeneity of studies including populations, CHD included and timings and methods of sample analysis prevented meta‐analysis. Therefore, the study focused on a narrative synthesis. Utilization of classifications of CHD such as those provided by EUROCAT and increased sample sizes with subgroup analyses could reduce heterogeneity and strengthen future findings (EUROCAT, 2013).

## 5 CONCLUSION

In conclusion, there is not enough evidence to confidently determine if maternal micronutrient deficiency increases the risk of CHD in fetuses. Further studies are required with prospective or nested case control design utilizing established micronutrient reference ranges ideally collecting samples in pregnancy and postnatally. The UK‐based Surgical‐PEARL (Surgical Paediatric congEnital Anomalies Registry with Long term follow‐up) multicenter cohort study has commenced recruitment in 2022 (https://www.isrctn.com/ISRCTN12557586). This will recruit fetuses with congenital anomalies potentially requiring surgery and their biological mothers and fathers at the time of diagnosis, collecting routine clinical and imaging data, postnatal surgical data and a range of fetal, childhood and parental biosamples. Through resources such as this and others, we aim to establish a greater evidence base to robustly answer this question.

## AUTHOR CONTRIBUTIONS

SM conceived and designed the study; acquired, analyzed and interpreted the data; and drafted and approved the manuscript. CS conceived and designed the study; acquired, analyzed and interpreted the data; and drafted and approved the manuscript. MC contributed to study design; and drafted and approved the manuscript. TO contributed to study design; and drafted and approved the manuscript.

## CONFLICT OF INTEREST

The authors declare no conflicts of interest.

## Supporting information


**Figure S1**Detailed search strategy for MEDLINE, EMBASE, and Cochrane Library.

**Table S1** Characteristics of included studies. ASD, atrial septal defect; AVSD, atrioventricular septal defect; AV, aortic valve; DORV, double outlet right valve; HLHS, hypoplastic left heart syndrome; LVOT, left ventricular outflow tract; PDA, patent ductus arteriosus; PV, pulmonary valve; RVOT, right ventricular outflow tract; TGA, transposition of the great arteries; ToF, tetralogy of fallot; VSD, ventricular septal defect. ^**†**^Methodology checklist classification derived utilizing the Scottish Intercollegiate Guidelines Network (SIGN) case control study risk of bias tool by two authors (SM and CS). Assessment of subject selection, exposure assessment, confounding and statistical analysis classifies studies overall as high quality (++), acceptable (+) or unacceptable (−).

## Data Availability

The data that support the findings of this study are available from the corresponding author upon reasonable request.
