# Supplementary material for: Maternal micronutrient deficiency and congenital heart disease risk: A systematic review of observational studies
Source: Birth Defects Res. 2022 Aug 18;114(17):1079–91. doi: 10.1002/bdr2.2072 (PMC9805156; doi:10.1002/bdr2.2072)
Supplement: Supplementary file 1 — Figure S1Detailed search strategy for MEDLINE, EMBASE, and Cochrane Library. [file BDR2-114-1079-s002.docx]

Supplementary Figure 1 (S1) – Search Strategies

**Ovid MEDLINE(R) <1946 to present>**

1 exp Heart Defects, Congenital/ 159088

2 Micronutrients/ 6518

3 Vitamins/ 32629

4 Deficiency Diseases/ 7924

5 exp Avitaminosis/ 76529

6 congenital heart disease*.tw. 29086

7 congenital cardiac disease*.tw. 703

8 congenital heart anomal*.tw. 339

9 congenital cardiac anomal*.tw. 768

10 maternal vitamin*.tw. 1234

11 maternal micronutrient*.tw. 135

12 maternal zinc.tw. 185

13 maternal copper.tw. 31

14 maternal iron.tw. 405

15 maternal fol*.tw. 607

16 1 or 6 or 7 or 8 or 9 168725

17 2 or 3 or 4 or 5 or 10 or 11 or 12 or 13 or 14 or 15 118578

18 16 and 17 262

**Embase <1974 to 2021 September 03>**

1 exp congenital heart malformation/ 139662

2 trace element/ 36979

3 vitamin/ 40634

4 nutritional deficiency/ 15080

5 exp vitamin deficiency/ 71693

6 congenital heart disease*.tw. 43251

7 congenital cardiac disease*.tw. 940

8 congenital heart anomal*.tw. 538

9 congenital cardiac anomal*.tw. 1147

10 maternal vitamin*.tw. 1736

11 maternal micronutrient*.tw. 163

12 maternal zinc.tw. 247

13 maternal copper.tw. 39

14 maternal iron.tw. 558

15 maternal fol*.tw. 840

16 1 or 6 or 7 or 8 or 9 164759

17 2 or 3 or 4 or 5 or 10 or 11 or 12 or 13 or 14 or 15 154182

18 16 and 17 531

**Cochrane Library**

ID Search Hits

#1 MeSH descriptor: [Heart Defects, Congenital] explode all trees 2318

#2 MeSH descriptor: [Micronutrients] this term only 988

#3 MeSH descriptor: [Vitamins] this term only 4189

#4 MeSH descriptor: [Deficiency Diseases] this term only 218

#5 MeSH descriptor: [Avitaminosis] explode all trees 2575

#6 congenital heart disease*.tw 2

#7 congenital cardiac disease*.tw 2

#8 congenital heart anomal*.tw 0

#9 congenital cardiac anomal*.tw 0

#10 maternal vitamin*.tw 1

#11 maternal micronutrient*.tw. 0

#12 maternal zinc.tw. 4

#13 maternal copper.tw. 0

#14 maternal iron.tw. 5

#15 maternal fol*.tw. 0

#16 #1 OR #6 OR #7 OR #8 OR #9 2320

#17 #2 OR #3 OR #4 OR #5 OR #10 OR #11 OR #12 OR #13 OR #14 OR #15 6950

#18 #16 AND #17 10
