# Supplementary material for: Maternal micronutrient deficiency and congenital heart disease risk: A systematic review of observational studies
Source: Birth Defects Res. 2022 Aug 18;114(17):1079–91. doi: 10.1002/bdr2.2072 (PMC9805156; doi:10.1002/bdr2.2072)
Supplement: Supplementary file 2 — Table S1 Characteristics of included studies. ASD, atrial septal defect; AVSD, atrioventricular septal defect; AV, aortic valve; DORV, double outlet right valve; HLHS, hypoplastic left heart syndrome; LVOT, left ventricular outflow tract; PDA, patent ductus arteriosus; PV, pulmonary valve; RVOT, right ventricular outflow tract; TGA, transposition of the great arteries; ToF, tetralogy of fallot; VSD, ventricular septal defect. † Methodology checklist classification derived utilizing the Scottish Intercollegiate Guidelines Network (SIGN) case control study risk of bias tool by two authors (SM and CS). Assessment of subject selection, exposure assessment, confounding and statistical analysis classifies studies overall as high quality (++), acceptable (+) or unacceptable (−). [file BDR2-114-1079-s001.docx]

| **Study Reference** | **Study Setting** | **Study Design** | **Exposure** | **Outcome** | **Relevant Inclusion Criteria** | **Relevant Exclusion Criteria** | **Methodology Checklist †** |
| --- | --- | --- | --- | --- | --- | --- | --- |
| **Dilli et al. 2018** | Turkey | Case Control  Cases n=108  Controls n=103 | Micronutrients  Vitamin B12  Folate  Vitamin A  Zinc  Vitamin D  Source  Blood, serum  Collection Timing  Postnatal | CHD Diagnosis Method  Clinical grounds, typical findings on chest radiograph, ECG.  Diagnosis confirmed echocardiography or catheterisation.  Diagnosis Timing  Before 1 month of age.  CHD Diagnoses  Coarctation aorta/Interrupted aortic arch 27.8%  Pulmonary atresia 14.8%  Tricuspid atresia 12.9%  HLHS 10.2%  TGA 9.2%  Critical pulmonary stenosis 9.2%  Fallot type pulmonary atresia 5.7%  VSD 5.7%  Other diagnoses 4.5% | Cases  Mothers of:  Babies born in hospital or transferred to neonatal intensive care unit.  Gestation birth 35-42 weeks.  Controls  Gestational age week of birth, postnatal age and sex matched to case. | Cases  CHD diagnosed >1 month age.  Both  Gestational age delivery <35 weeks or >42 weeks. | + |
| **Verkleij-Hagoort et al. 2006** | Netherlands | Case Control  Cases n=98  Controls n=134 | Micronutrients  Red blood cell folate  Serum folate  Vitamin B12  Source  Blood, venous and serum  Collection Timing  11-18 months postnatal | CHD Diagnosis Method  Echocardiography and/or cardiac catheterisation and/or surgery.  Diagnosis Timing  After birth  CHD Diagnoses  VSD 29.1%  Pulmonary stenosis 19.9%  TGA 18.5%  Perimembranous  Tetralogy of Fallot 12.6%  Coarctation aorta 9.3%  AVSD 6.0%  AV stenosis 3.3%  HLHS 1.3% | Cases  Mothers of:  Children with CHD diagnosed as described. | Controls  Familial relationship to cases  Any major anomalies or chromosomal abnormality. | + |
| **Hobbs et al. 2005** | USA | Case Control  Cases n=224  Controls n=90 | Micronutrients  Plasma folate  Vitamin B12  Source  Blood, plasma  Collection Timing  Postnatal; median 14.9 months case, 24.5 months control since pregnancy. | CHD Diagnosis Method  Physician diagnosis by prenatal or postnatal echocardiography, surgical or autopsy report.  Diagnosis Timing  Identification through state birth defect registry.  CHD Diagnoses  Septal, conotruncal or obstructive right or left sided defect. | Cases  Mothers of:  Pregnancy outcome was a liveborn, stillborn, or elective termination.  Controls  Live births with no birth defect.  Both  Case and control subjects had completed participation in the National Birth Defects Prevention Study. | Cases  Syndromic, single gene or chromosomal abnormality.  Both  Pregnant at time of blood sample.  On antiepileptic medications at time of blood sample. | ++ |
| **Hu et al. 2014** | China | Case Control  Cases n=212  Controls n=212 | Micronutrients  Copper  Zinc  Source  Hair  Collection Timing  Not specified | CHD Diagnosis Method  Echocardiography.  Diagnosis Timing  Antenatally with fetal echocardiography, confirmed within 3 months of birth.  CHD Diagnoses  Conotruncal 41.98%  Septal 25%  RVOT obstruction 23.58%.  LVOT obstruction 23.11%.  Other 8.96%  Anomalous pulmonary venous return 2.83%.  Some patient >1 anomaly. | Cases  Pregnant women with fetal CHD diagnosed on echo >14/40, livebirths, stillbirths or TOP  Control  Pregnant woman with no fetal abnormality, same hospital, livebirths. | Both  Folate antagonists and AEDs, CHD antenatally but not confirmed postnatally, single umbilical artery, dysrhythmia or isolated cardiomyopathy, genetic syndrome or unclear diagnosis, extracardiac malformations, multiple birth, hair dye in mother, mother mental disorder. | ++ |
| **Ou et al. 2017** | China | Case Control  Cases n=112  Controls n=107 | Micronutrients  Copper  Selenium  Source  Blood, plasma  Collection Timing  17-40 weeks | CHD Diagnosis Method  Echocardiography.  Diagnosis Timing  Fetal echocardiography and confirmed postnatally.  CHD Diagnoses  Septal 55.4%  Conotruncal 37.5%  RVOT obstruction 28.6%  LVOT obstruction 14.3%.  Other 9.8%%  Some patient >1 anomaly. | Controls  Matched gestational age within 3 months, maternal age within 5 years, routine prenatal consultation | Cases  Only mitral or tricuspid valvular defects or enlarged ASDs  Chromosomal malformation or syndromes, prenatal CHD not confirmed after delivery.  Controls  Family history of CHD, diabetes, multiple pregnancy. | ++ |
| **Koster et al. 2018** | Netherlands | Case Control  Cases n=345  Controls n=432 | Micronutrients  25-OH Vitamin D  Source  Blood, serum  Collection Timing  15 months postnatal | CHD Diagnosis Method  Method not specified. Recruitment from public child health centers monitoring development of all children < 4 years.  Diagnosis Timing  Paediatric cardiologist.  CHD Diagnoses  Perimembranous VSD 29.3%  TGA 16.5%  PV Stenosis 14.8%  ToF 13.3%  Coarctation aorta 10.4%  AVSD 10.1%  HLHS 4.3%  Other 3.5%  AV stenosis 2% |  | Both  Lactating mothers | + |
| **Mokhtar et al. 2019** | Egypt | Case Control  Cases n=50  Controls n=50 | Micronutrients  25-OH Vitamin D  Source  Blood, serum  Collection Timing  Not specified | CHD Diagnosis Method  Echocardiography.  Diagnosis Timing  1^st^ 2 weeks of life.  CHD Diagnoses  VSD 28%  AAD 22%  PDA 18%  TGA 10%  ToF 6%  Atrioventricular defect 6%  Trucus arteriosus 4%  HLHS 4%  DORV 2%  Some patient >1 anomaly. | Controls  healthy mothers with age and sex harmonised neonates with no CHD. | Both  neonatal sepsis, congenital infection, genetic syndromes or multiple anomalies, maternal diabetes, hypertension, autoimmune | + |
| **Yang et al. 2020** | China | Case Control  Included  Cases n=474  Controls n=948  Nutritional Analysis  Cases n=50  Controls n=100 | Micronutrients  Ferritin  Source  Blood, serum  Collection Timing  While waiting for delivery | CHD Diagnosis Method  Antenatal ultrasound confirmed by physical examination after birth.  Diagnosis Timing  Fetal.  CHD Diagnoses  VSD 46.8%  ASD 46.0%  AVSD 18.3%  PDA 15.6%  Proportions of total cases  Some patient >1 anomaly. | Controls  no diagnosed anomalies, same month, same hospital 2:1 | Both  Diabetes, multiple gestations, gene disorders, chromosomal anomalies | + |
